# Astley Cooper Prize

**Published:** 1850-07

**Authors:** 


ASTLEY COOPER PRIZE.

The following communication was sent to us by a valued friend, with the suggestion that it should be published in the Examiner, for the benefit of aspirants in this country. It was unfortunately mislaid until the moment of going to press. We have, therefore, merely the opportunity of laying it before our readers, without comment.—[EDITOR MEDICAL EXAMINER.

The subject for the fourth triennial prize of three hundred pounds, under the will of the late Sir Astley Cooper, Bart., is "*The Structure and use of the Spleen.*"

The condition annexed by the Testator is, "That the Essays or Treatises written for such prize shall contain original experiments and observations, which shall not have been previously published; and that such Essays or Treatises shall (as far as the subject shall admit of) be illustrated with drawings, which preparations and drawings shall be added to the Museum of Guy's Hospital and shall, together with the work itself and the sole and exclusive interest therein and the copyright thereof, become thenceforth the property of the Hospital, and be transferred as such by the successful candidate."

It is the will of the Founder that no Physician, or Surgeon, or other officer, for the time being, of Guy's Hospital or St. Thomas' Hospital, nor any person related by blood or affinity to any such Physician or Surgeon, or other officer for the time being, shall at any time be entitled to claim the prize; but, with the exception here referred to, this (the Astley Cooper) Prize is open for competition to the whole world.

Candidates are informed that their Essays, either written in the English Language, or, if in a Foreign Language, accompanied by an English translation, must be sent to Guy's Hospital on or before January 1st, 1853, addressed to the Physicians and Surgeons of Guy's Hospital.

Each Essay or Treatise must be distinguished by a Motto, and be accompanied by a sealed envelope containing the name and address of the Writer. None of the sealed envelopes will be opened, except that which accompanies the successful Treatise. The unsuccessful Essays and Treatises, with the illustrative preparations and drawings, will remain at the Museum of Guy's Hospital until claimed by the respective writers or their agents.

